# Indoor thermal comfort in a rural dwelling in southwest China

**DOI:** 10.3389/fpubh.2022.1029390

**Published:** 2022-09-29

**Authors:** Dong Wei, Guilin Zhao, Sheng Liu, Linchuan Yang

**Affiliations:** School of Architecture, Southwest Jiaotong University, Chengdu, China

**Keywords:** thermal sensation vote, thermal adaptive behavior, questionnaire survey, operative temperature, rural area, hot-summer and cold-winter region

## Abstract

Recently, indoor thermal comfort has received more scholarly attention than ever due to the COVID-19 pandemic and global warming. However, most studies on indoor thermal comfort in China concentrated on urban buildings in the east and north. The indoor thermal comfort of rural dwellers in southwest China is insufficiently investigated. Hence, this study assesses residents' indoor thermal comfort in a rural dwelling in Linshui, obtains the thermal neutral temperature of the rural area, and analyzes the thermal adaptation behavior of rural dwellers. The results reveal that the thermal neutral temperature of rural dwellers is 29.33°C (operative temperature), higher than that presented in previous studies based on the same climate region. Indoor thermal conditions in rural dwellings are relatively harsh, but various thermal adaptation behavior of rural dwellers significantly improve their ability to withstand the harsh conditions. When people live in an environment with a (relatively) constant climate parameter (e.g., humidity), their perception of that parameter seems compromised. Most rural dwellers are unwilling to use cooling equipment with high energy consumption. Therefore, more passive cooling measures are recommended in the design and renovation of rural dwellings.

## Introduction

In the context of global warming, extreme heat events have been frequently observed. Some studies demonstrate that extreme heat events pose a serious threat to human health, with excessive temperatures causing subhealth symptoms (e.g., fatigue, dizziness, tachypnea, and tachycardia) and that they are even life-threatening and fatal in severe cases (1–3). Therefore, creating a comfortable indoor thermal environment is essential to deal with excessive temperatures, reduce energy consumption, and safeguard residents' health (e.g., preventing heat-related illnesses such as heat exhaustion) (4–6).

The majority of people spend over 80% of their time indoors (7), so the quality of the indoor environment, which is essential for their physical and psychological health (8–10), is essential. Moreover, the American Society of Heating, Refrigerating and Air-Conditioning Engineers (ASHRAE) defines thermal comfort as “the condition of mind that expresses satisfaction with the thermal environment.” Thus, thermal comfort is normally evaluated subjectively. Furthermore, the importance of whether people feel comfortable with the indoor thermal environment has recently been emphasized (11, 12). During the COVID-19 pandemic, people spent more time indoors than ever because of mandatory quarantine, stay-at-home orders, travel restrictions, and community closure (13–15). Therefore, indoor thermal comfort should receive much more scholarly attention in this era.

A goal of sustainable development is to design and build dwellings with a comfortable indoor thermal environment without increasing energy consumption. The thermal environment is influenced by various factors, such as wall materials (16–19), the outdoor environment (20–26), and active equipment (27, 28). Numerous locations have devised new construction and renovation solutions to alter the indoor thermal environment of dwellings by changing the above factors (29–31). However, in the rural areas of developing countries, the application and promotion of these (perhaps expensive) design and construction techniques are limited because of poor architectural design technologies and underdeveloped economic conditions (8), thereby posing severe threats to the indoor comfort and health of rural dwellers. China is a predominantly agricultural country with a large number of rural dwellers. According to data released by the National Bureau of Statistics in February 2022 (http://www.stats.gov.cn/), as of the end of 2021, 498.35 million people, which accounted for 35.3% of the total population, lived in rural areas. Therefore, enhancing indoor thermal comfort in rural dwellings in China should receive great attention. An essential step in improving the quality of life and health of rural dwellers is to understand their indoor thermal comfort.

Most studies on indoor thermal comfort aim to understand residents' satisfaction with the indoor thermal environment and, consequently, to improve the quality of their indoor environment. The results of such studies can help improve residents' health and living environment (32, 33). The goals of such studies are in line with the United Nations' Sustainable Development Goals (SDGs), such as SDG 3 “good health and well-being” and SDG 11 “sustainable cities and communities.” As of today, numerous studies have focused on indoor thermal comfort in many locations, such as the USA (34, 35), Europe (36–38), and Australia (39, 40). In the context of China, researchers mainly focused on the northeast (41, 42), western (43, 44), and coastal (5, 45–47) regions. Comparatively, indoor thermal comfort in rural dwellings in southwest China received limited attention, although some studies on urban buildings in this region exist (48). Consequently, this study chooses a rural dwelling in Linshui County, southwest China, as the case to explore the indoor thermal comfort of rural dwellers by using subjective and objective methods (32).

The contributions of this paper can be summarized as below: [1] focusing on an under-studied location (a rural area in southwest China) and diversifying the focus of the existing literature on human thermal comfort; [2] examining rural dwellers' perceptions and preferences of indoor thermal environment parameters and analyzing the changes in their perceptions because of long-term adaptation; [3] determining the thermal neutral temperature of rural areas in a hot-summer and cold-winter (HSCW) region of China; and [4] identifying rural dwellers' thermal adaptation behavior patterns. This study provides a valuable reference for rural dwelling design, contributing to optimizing the indoor thermal environment and enhancing the health of rural dwellers.

## Methodology

### Climate classification in China

**Figure 1:**
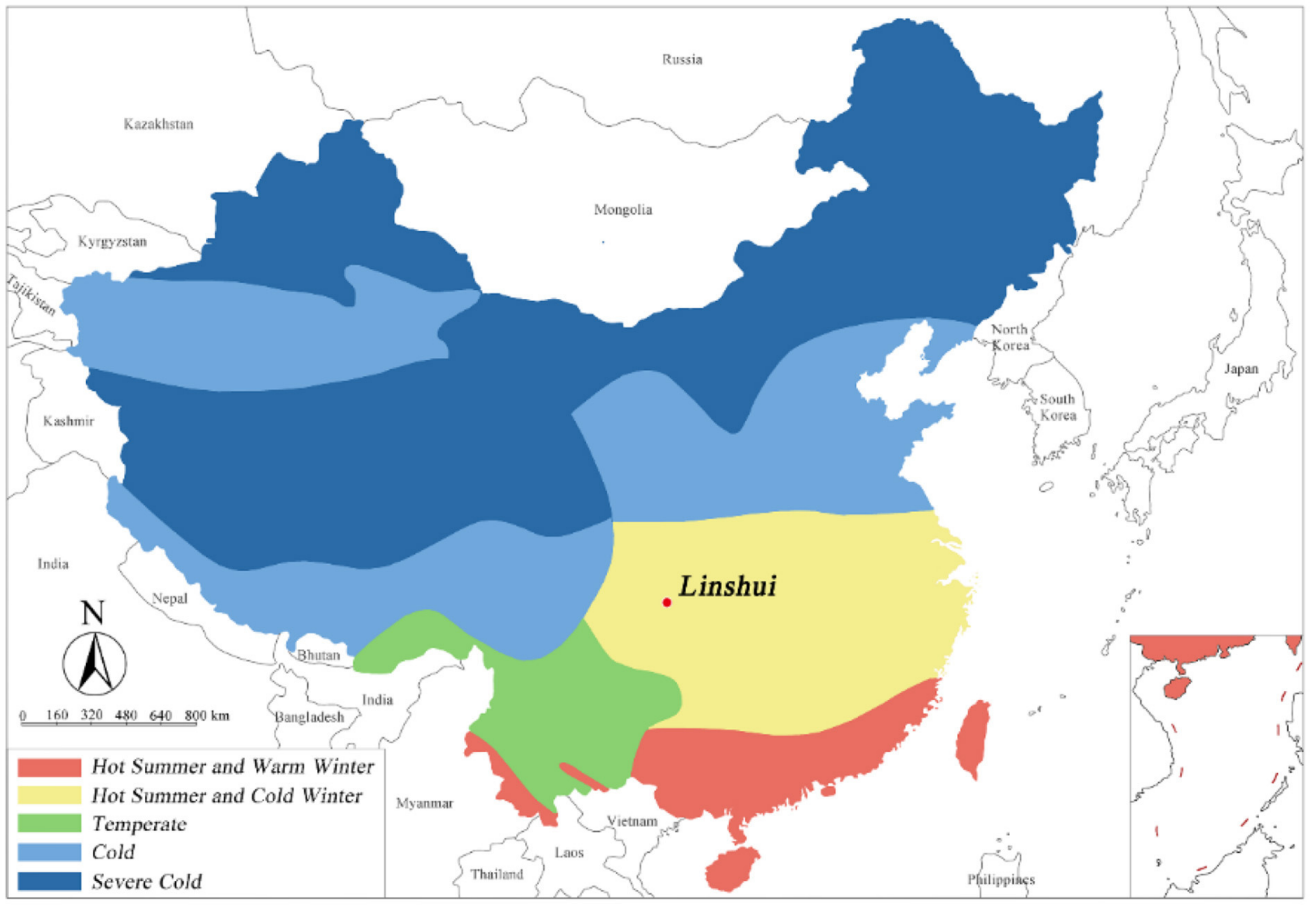
Climate region for building thermal design of China. Adapted from the Code for Thermal Design of Civil Buildings, GB 50176–2016.

China is the third-largest country in the world. It has a highly diverse and complex climate, leading to the formation of numerous regions with diverse climatic characteristics. China has formulated the Code for Thermal Design of Civil Buildings (GB 50176–2016). The code divides China into five climate regions based on the average temperatures of the coldest and warmest months of a year: severe cold (SC) region, cold (C) region, hot-summer and warm-winter (HSWW) region, HSCW region, and temperate (T) region (see Figure 1).

The rural dwelling assessed in this study is located in an HSCW region, where the average temperatures of the coldest and warmest months are 0–10 and 25–30°C, respectively.

### Study area

**Figure 2:**
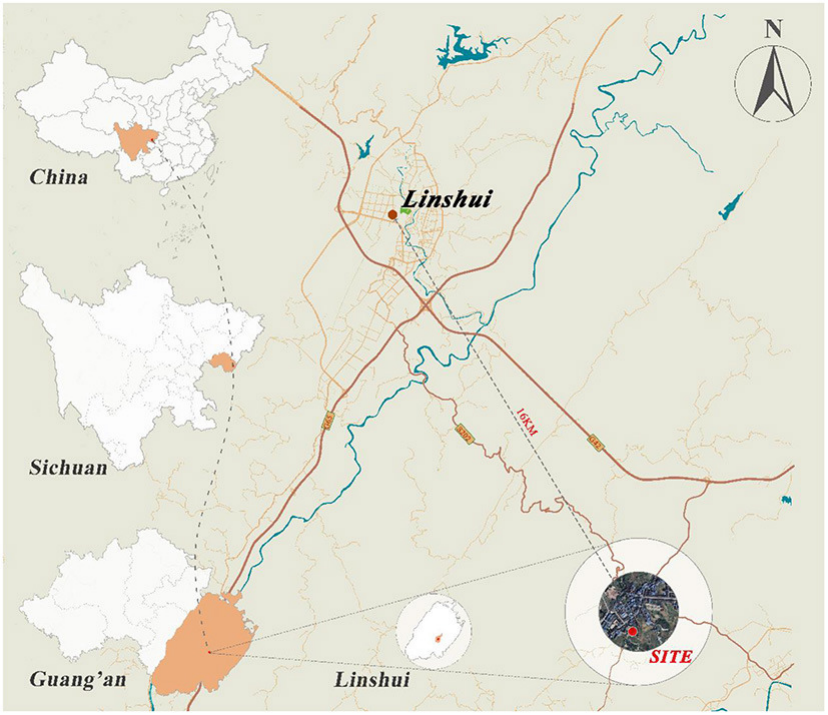
The location of Linshui. Elaborated by the authors.

Linshui is a county in Guang'an, northeast Sichuan, southwest China (Figure 2). It is the county closest to Chongqing in Sichuan Province and is 90 km from the urban district of Chongqing. Its longitude ranges from 106°41^′^ to 107°18^′^E, while its latitude ranges from 30°01^′^ to 30°33^′^N. Linshui has an area and altitude of 1,909 km^2^ and 366 m, respectively. As of December 2020, the population was 1.00 million, with an urbanization rate of 21.31%.

**Figure 3:**
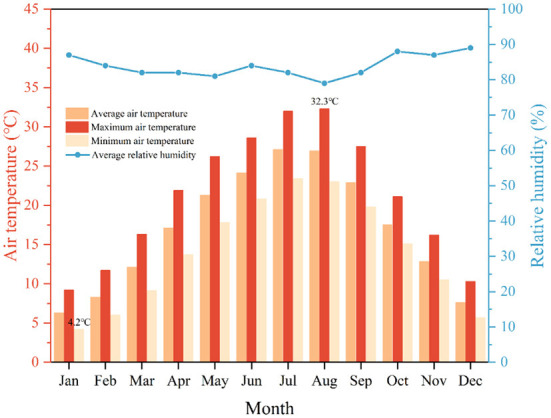
Monthly average, maximum, and minimum ***T***_***a***_ and ***RH*** in Linshui from 1981 to 2010. Elaborated by the authors using the data from the National Meteorological Science Data Center, website: http://data.cma.cn/.

Linshui has a humid subtropical climate with mild winters, abundant rainfalls, and long summers. Figure 3 shows the monthly average, maximum, and minimum air temperatures (*T*_*a*_) and average relative humidity (*RH*) of Linshui between 1981 and 2010. The highest and lowest temperatures are observed in August and January, respectively. The annual average temperature difference is about 20°C. Summer is hot in Linshui, and July and August are high-temperature seasons. The average *RH* is stable, with around 10% variance between the maximum and minimum.

### Field measurements

**Figure 4:**
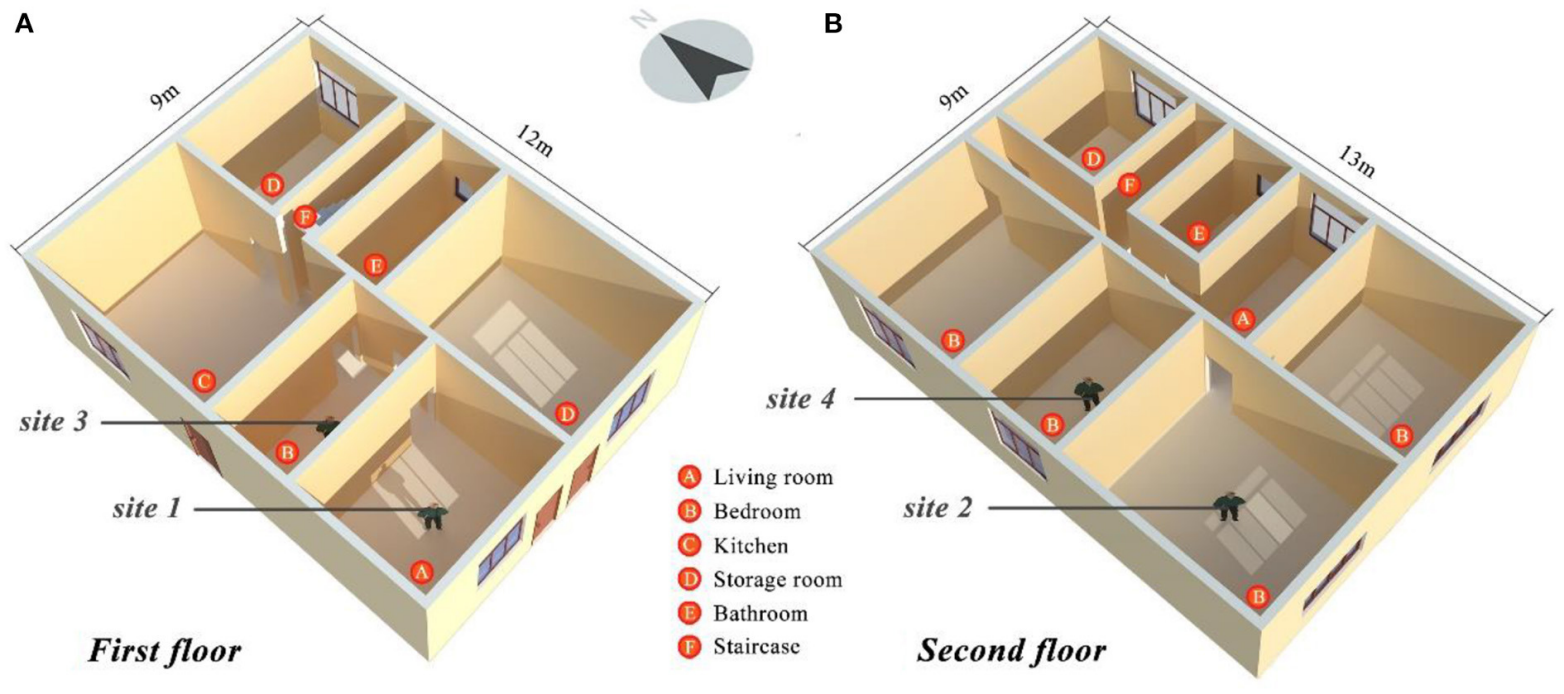
The layout of rural dwellings and the location of the measurement sites **(A)** First Floor, **(B)** Second Floor.

Objective measurements and subjective questionnaires were conducted during the hottest month (August) to assess indoor thermal comfort in a rural dwelling in Linshui (Figure 4). The chosen rural dwelling (30°12^′^13″N, 107°00^′^47″E) was built in 2002. It is a two-story house with a total area of 225 m^2^. The first and second floors are 3.8 and 3.0 m high, respectively. Multiple (four) sites were chosen for measurement, enabling the comprehensive monitoring of the thermal comfort of residents who spend most of their time in living rooms and bedrooms.

Field measurements were conducted between 21:00 August 3 and 17:15 August 6, 2019. Indoor *T*_*a*_, *RH*, black globe temperature (*T*_*g*_), and air velocity (*v*) were assessed using the measuring instrument listed in Table 1. The measurement tool is installed at each measurement location. The detector is placed 1.5 m above the ground, collecting the data at an interval of 15 min. The air-conditioner is off in the room during the measured period. All parameters (range, accuracy, and resolution) of the instruments used for the study meet the requirement of the ASHRAE Standard 55–2017 (21).

**Table 1:** Specifications of the measuring instrument used in this study.

**Instrumentation**	**Parameters**	**Range**	**Accuracy**	**Resolution**	**Photos**
Testo, 174H-Mini	Air temperature (***T**_***a***_*)	−20–70°C	±0.5°C	0.1°C	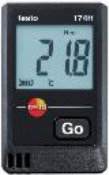
	Relative humidity (***RH***)	0–100%	±3%	0.1%	
JT TECH., JRT04	Globe temperature (***T**_***g***_*)	−20–125°C	±0.2°C	0.1°C	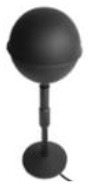
TENMARS, TM-404	Air velocity (***v***)	0–25 m/s	±2%	0.01 m/s	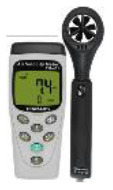

### Questionnaire survey

**Figure 5:**
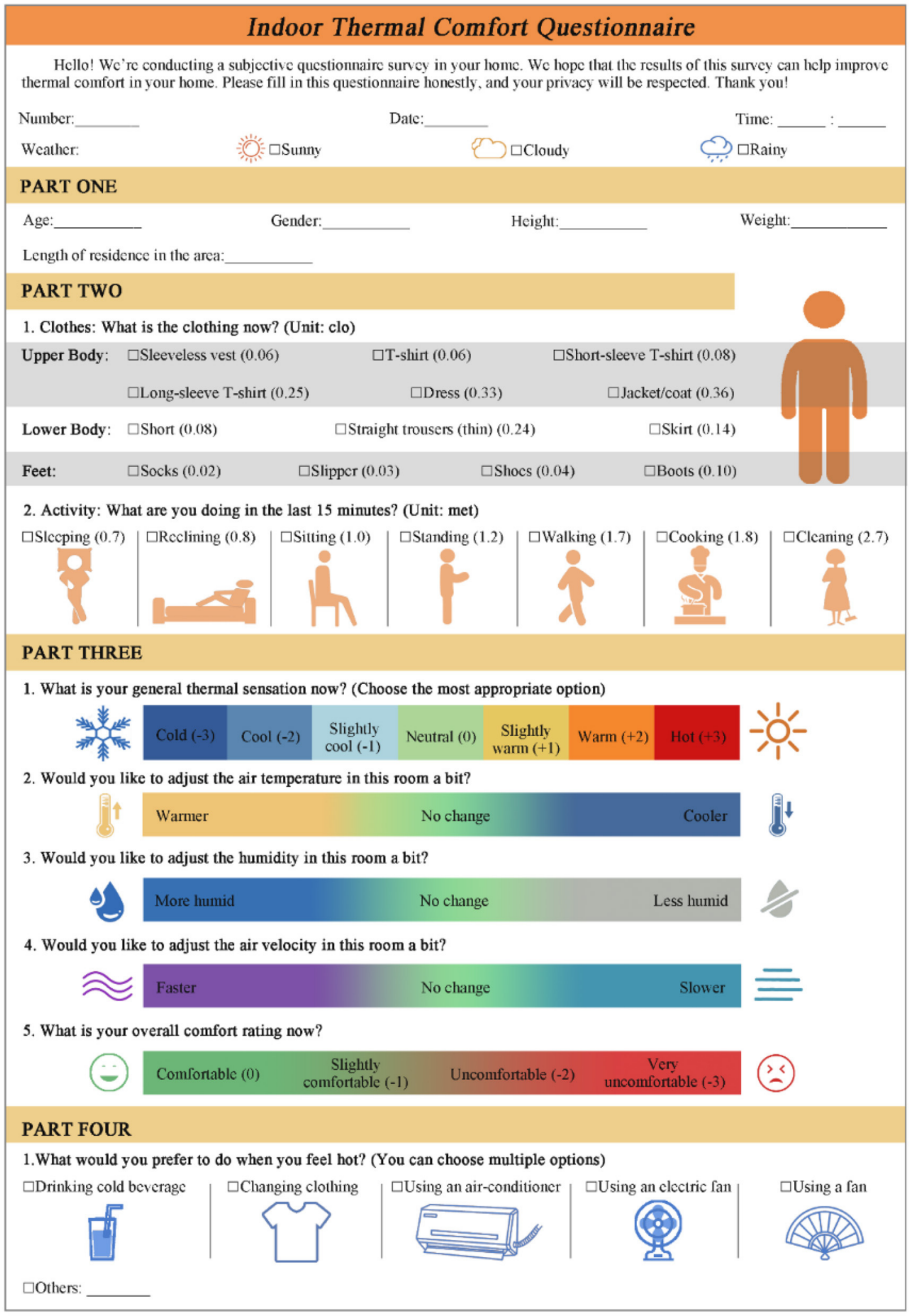
The subjective survey questionnaire used.

Figure 5 shows the four-part subjective questionnaire. The respondents are rural dwellers living nearby. The number of respondents is smaller than the number of questionnaires because of repeated observations.

Following a face-to-face interview, the interviewer will complete the first part by asking the respondent for basic personal characteristics (i.e., age, sex, height, weight, and length of residence).

The second part records the clothing insulation (unit: clo) and activity (unit: met) status of the respondent.

The third part collects the respondent' subjective responses to five questions on their indoor thermal perception, preference for indoor thermal environment parameters (*T*_*a*_, *RH*, and *v*), and overall thermal comfort. The respondents must evaluate the corresponding options on an ASHRAE 7-point scale and select their answer based on their current thermal sensation when answering question 1. This is known as thermal sensation voting (TSV). Questions 2–4 are preference surveys of indoor thermal environment parameters using 3-point scales to study the respondents' most sensitive environmental parameters. Question 2 is defined as air temperature preference vote (TPV), question 3 is defined as relative humidity preference vote (HPV), and question 4 is defined as air velocity preference vote (VPV). Question 5 is the questionnaire validity test question. It is defined as the overall comfort vote (OCV). The questionnaire is considered to be valid if the results of the TSV and OCV are identical. Table 2 indicates the relationship between the vote results for TSV and OCV.

**Table 2:** Relationship between the TSV and OCV.

**Value of OCV**	**−3**	**−2**	**−1**	**0**
**Value of TSV**	**+3 or −3**	**+2 or −2**	**+1 or −1**	**0**
**Result of TSV**	**Hot or cold**	**Warm or cool**	**Slight warm or slight cool**	**Neutral**
**Result of OCV**	**Very uncomfortable**	**Uncomfortable**	**Slightly comfortable**	**Comfortable**

The fourth part involves gathering information on respondents' summer thermal adaptation behavior to better understand how people in rural areas live.

### Data analysis

This study selects ***T***_***op***_ (operative temperature) as an index for evaluating the thermal comfort of rural dwellers. The indoor ***T***_***op***_ is calculated as follows:


(1) $$\begin{array}{l} T_{\text{op}}=A\times T_{a}+\left(1-A\right)\times T_{mrt}, \end{array}$$

where *T*_*mrt*_ is the mean radiant temperature (unit: °C), and it is calculated using Eq. (2). *A* can be selected from the following values (Table 3).

**Table 3:** Values of *A* that vary with *v*.

***v***	**<0.2 m/s** **(<40 fpm)**	**0.2 to 0.6 m/s** **(40 to 120 fpm)**	**0.6 to 1.0 m/s** **(120 to 200 fpm)**
***A***	**0.5**	**0.6**	**0.7**

*T*_*mrt*_ is calculated using *T*_*a*_, *T*_*g*_, and *v* of the indoor environment. *T*_*g*_ is measured using a black globe thermometer. The formula of *T*_*mrt*_ is shown as follows:


(2) $$\begin{array}{l} T_{mrt}={\left[{\left(T_{g}+273\right)}^{4}+\frac{1.10\times 10^{8}v^{0.6}}{\varepsilon D^{0.4}}\right]}^{0.25}-273, \end{array}$$

where *D* is globe diameter (=150 mm in this study) and ***ε*** is emissivity (=0.95).

## Result

### Summary of the objective measurement

As noted above, indoor thermal environment monitoring was conducted in a rural dwelling in Linshui, Sichuan, from August 3 to 6, 2019. *T*_*a*_, *RH, T*_*g*_, and *v* are directly collected by sensors. *T*_*mrt*_ and *T*_*op*_ are calculated using the above formulas with the input of the collected data. The characteristics of the indoor thermal environment for each site throughout the measured period were summarized in Table 4.

**Table 4:** Summary of the indoor thermal environment during the measured period.

Site	***T_a_*** **(°C)**			***RH*** **(%)**	***V_a_* (m/s)**	***T_g_* (°C)**	***T_mrt_* (°C)**	***T_op_* (°C)**
	**Avg.**	**Max.**	**Min.**	**Avg.**	**Avg.**	**Avg.**	**Avg.**	**Avg.**
Site 1	28.24	34.50	24.60	82.82	1.21	29.27	30.90	29.77
Site 2	29.68	38.20	25.30	78.26	1.91	31.96	37.79	32.13
Site 3	29.23	30.70	27.40	80.41	0.09	29.95	30.32	29.78
Site 4	29.81	31.70	28.20	73.54	0.81	30.49	31.73	30.46
Total	29.24	-	-	78.76	1.01	30.53	33.19	30.54

The average *T*_*a*_ for the entire dwelling is 29.24°C. It was a reasonably high value, much higher than the average *T*_*a*_ for the whole county in August (26.9°C) (see Figure 3). In addition, the average *T*_*a*_ of the four sites was very close (the range = 1.57°C). These observations provide some evidence of global warming (49, 50).

Site 2 had the largest window-to-wall ratio among the four sites. Thus, it received the most solar radiation in the middle of the day, resulting in a rapid increase in temperature. Consequently, site 2 had the highest temperature (38.2°C) of the four sites.

No discernible variation in ***RH*** was noted across the four sites. The average *RH* of the entire dwelling was 78.76%, which is essentially the same as the average *RH* of the whole county in August (see Figure 3). Moreover, regarding *v*, sites 2 and 3 were the largest and smallest among the four sites, respectively. The difference was that site 2 had the maximum window space, directly impacted by the superficial air velocity, while site 3 had no windows and was essentially isolated from the outside environment. Site 2 had the highest *T*_*g*_, *T*_*mrt*_, and *T*_*op*_ average values across the four sites. This observation showed that enormous windows were more vulnerable to solar radiation, which impacted indoor thermal comfort.

**Figure 6:**
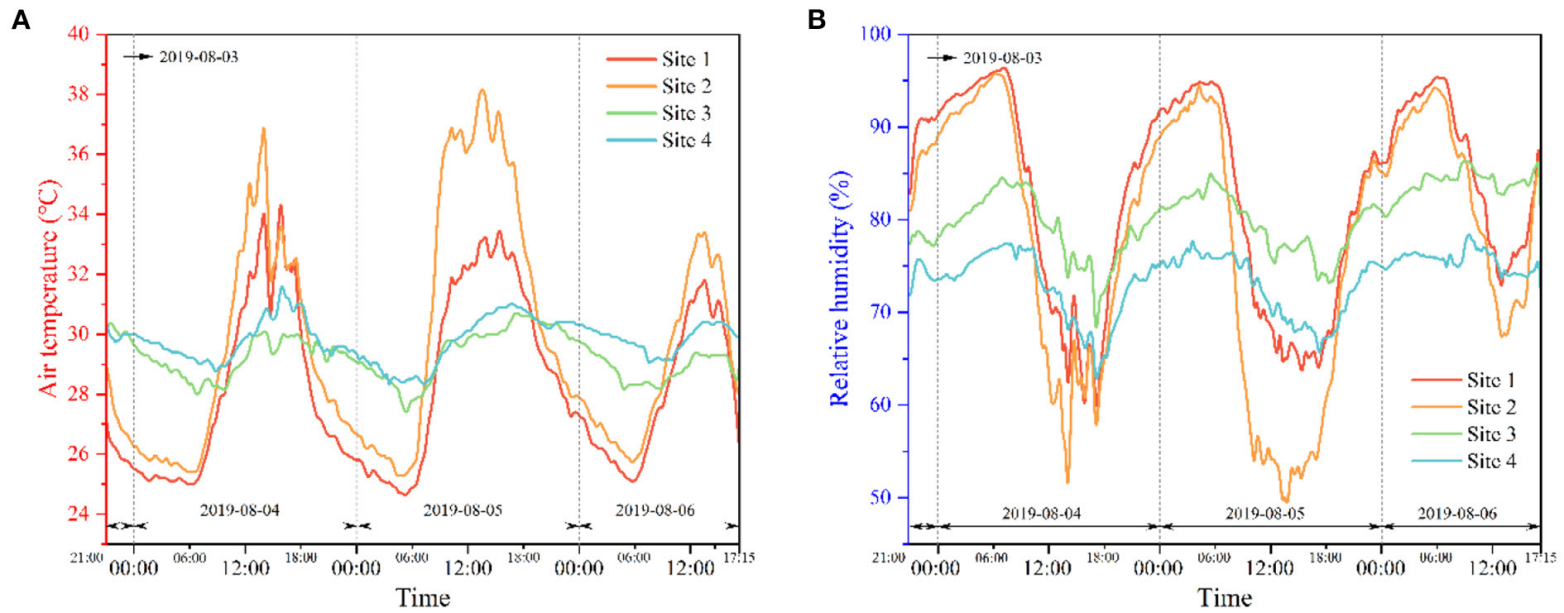
Comparison of T_a_ and *RH* at four measuring sites during the measured period **(A)** T_a_, **(B)** *RH*.

Figure 6 shows the *T*_*a*_ and *RH* data from the four sites. Sites 1 and 2 as well as sites 3 and 4 had highly similar trends. The reason is that the indoor environment conditions at sites 1 and 2 as well as sites 3 and 4 were similar.

From 9:00 to 18:00 on August 5, the temperature of site 2 was significantly higher than that of site 1, but the humidity of site 2 was significantly lower than that of site 1. The reason for this observation is that site 2 had closed windows and doors during this period. In addition, curtains were not used. Hence, the space was exposed to bright sunlight, resulting in a considerable temperature increase. Furthermore, the moisture in the room rapidly evaporated because of the increase in temperature, resulting in a restricted drop in humidity.

The trends of temperature and humidity at sites 3 and 4 were very similar. The temperature in site 4 was usually slightly higher than that in site 3. However, in terms of humidity, site 3 maintained a higher status than site 4. Site 3 had no windows and could not receive solar radiation. Thus, its temperature was lower than that of site 4, but the reverse was true for humidity. This phenomenon demonstrates that rooms with windows obtained a certain amount of solar radiation and affected temperature and humidity fluctuations directly and indoor thermal comfort indirectly. In general, solar radiation is a natural factor affecting the modification of indoor thermal environment characteristics and an indirect factor affecting the human body's thermal comfort.

### Summary of the subjective survey

This study collected 216 valid questionnaires. We summarize the respondents' basic personal information, clothing insulation, and activity status (metabolic rate). Females (59.72%) outnumbered males because most males in rural areas chose to migrate to cities to pursue a job, while females chose to raise their children at home. Men (58.3 years) had, on average, an older age than women (42.1 years) because older men had a compromised capability to work in distant cities and chose to stay in rural areas, while many young women stayed in rural areas for childbirth and child-rearing. All respondents have lived in the region for over 10 years. Thus, their thermal comfort is representative. Men's clothing insulation was lighter and thinner than women's, and their clothing insulation was typical of summer indoor dressing in terms of thermal resistance. Regarding activity status, the average metabolic rate for both men and women was 1.1 met, as most people were sitting indoors.

### Thermal sensation

**Figure 7:**
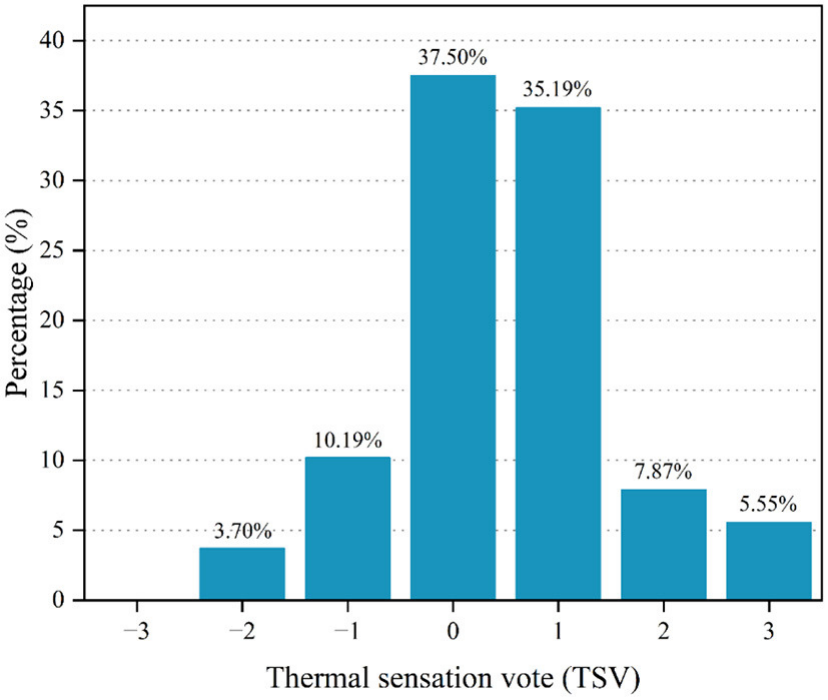
Result of thermal sensation vote.

The thermal sensation is a crucial psychological indicator of comfort or discomfort. This study aims to assess the various thermal responses of residents. Subjective assessments are necessary to evaluate the indoor thermal comfort of residents (32). The TSV distribution is shown in Figure 7. Most respondents (37.50%) indicated neutral (0) thermal sensation, with 10.19 and 35.19% of respondents voting −1 (slightly cool) and 1 (slightly warm), respectively. Respondents who voted 0, −1, and 1 were often considered comfortable with their current environment. Thus, 82.88% (0, 37.50%; −1, 10.19%; and 1, 35.19%) of the respondents considered themselves comfortable in summer, indicating that rural dwellers of Linshui adapted well to the local summer climate. The overall vote results of the respondents were on the hotter side, which was in line with the basic logic and confirmed that the questionnaire design of this study was reasonable. The analysis results of the data collected by the questionnaire were reasonable.

**Figure 8:**
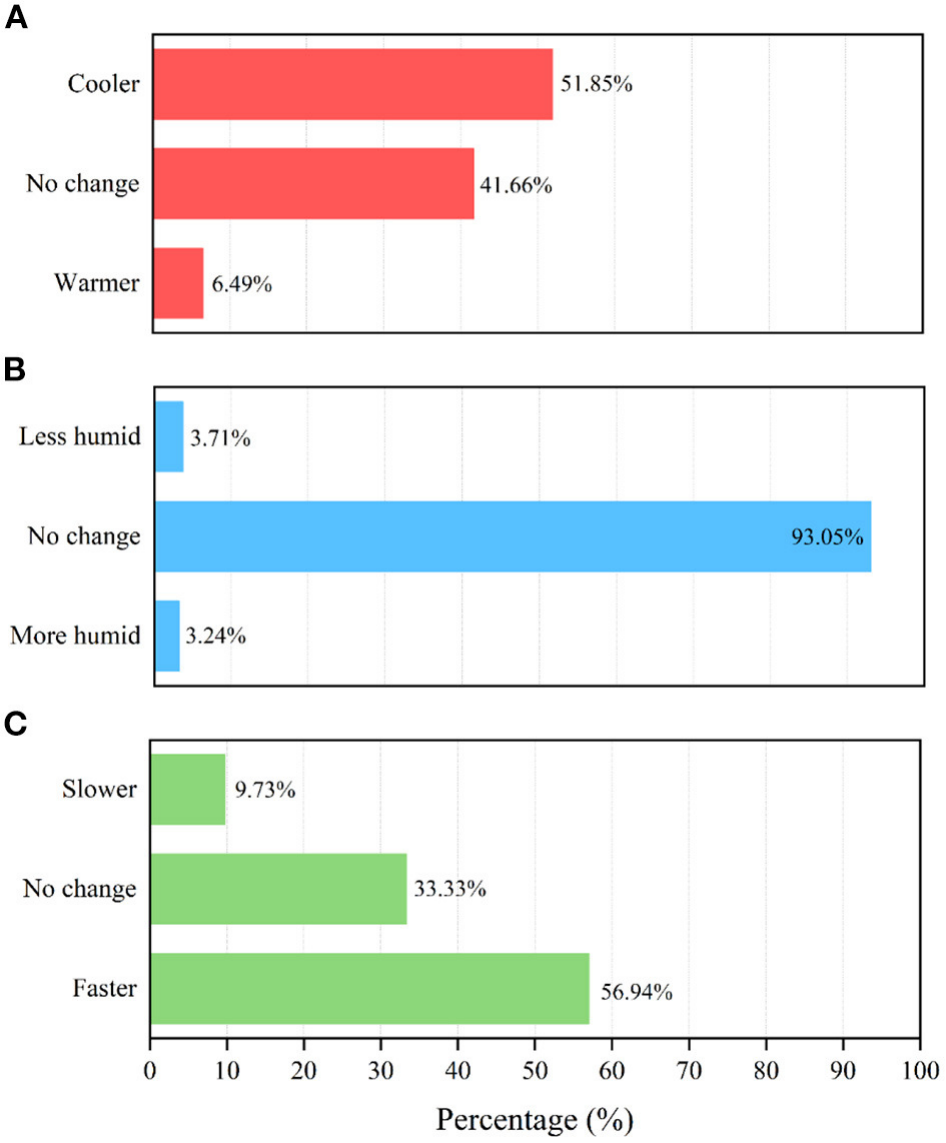
Results of indoor thermal environment parameter preference voting. **(A)** TPV, **(B)** HPV, **(C)** VPV.

Figure 8 reveals the results of indoor thermal environment parameter preference voting. The highest percentage of voting was *cooler* (51.85%), and the second-highest percentage was *no change* (41.66%) in terms of the TPV. Comparing the TSV results in Figure 7, we find that the percentage of those who voted −2 (cool) or −1 (slightly cool) was 13.89%, while the percentage of those who voted *warmer* in the TPV was 6.49%. This finding indicates that most respondents favored the cooler side in terms of temperature. Regarding HPV, the percentage of those who voted *no change* was very high, reaching 93.05%. This observation indicated that the residents do not perceive humidity changes significantly. In terms of the VPV, *faster* had the highest percentage of votes, and *no change* had the second-highest percentage of votes. The percentage of people who expected the air velocity to change was 66.67% (faster, 56.94%; slower, 9.73%), which means that most people could perceive the change in the air velocity.

Some phenomena worthy of attention were found from the voting results of indoor thermal environment parameter preference. Rural dwellers in Linshui perceived temperature and air velocity but had little perception of humidity.

**Figure 9:**
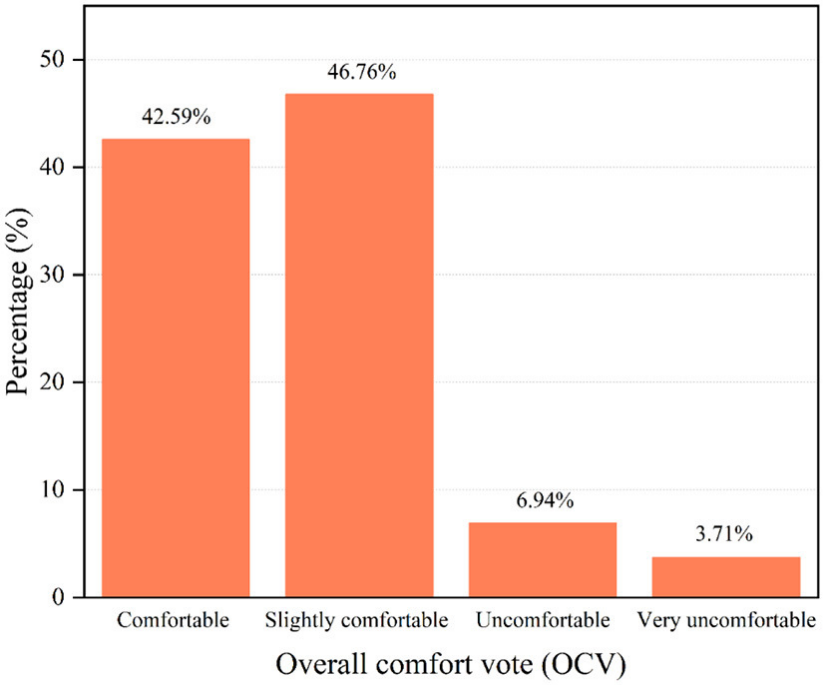
Result of the OCV.

Figure 9 shows the percentages of OCV in the studied rural dwelling. Analyzing the OCV can help validate the reasonableness of sensory heat voting fully. The TSV voting result of *neutral* (0) means that the patients feel comfort (0). The TSV voting result of *slightly warm* (1) or *slightly cool* (-1) means that they feel *slightly uncomfortable* (-1). According to the OCV results, 89.35% voted *comfortable* and *slightly comfortable* (comfort, 42.59%; slightly comfortable, 46.76%). The result of the TSV and OCV was very similar (89.35 vs. 82.88%). This finding again indicates that the questionnaire design of this study was adequate and that the collected data was reasonable.

**Figure 10:**
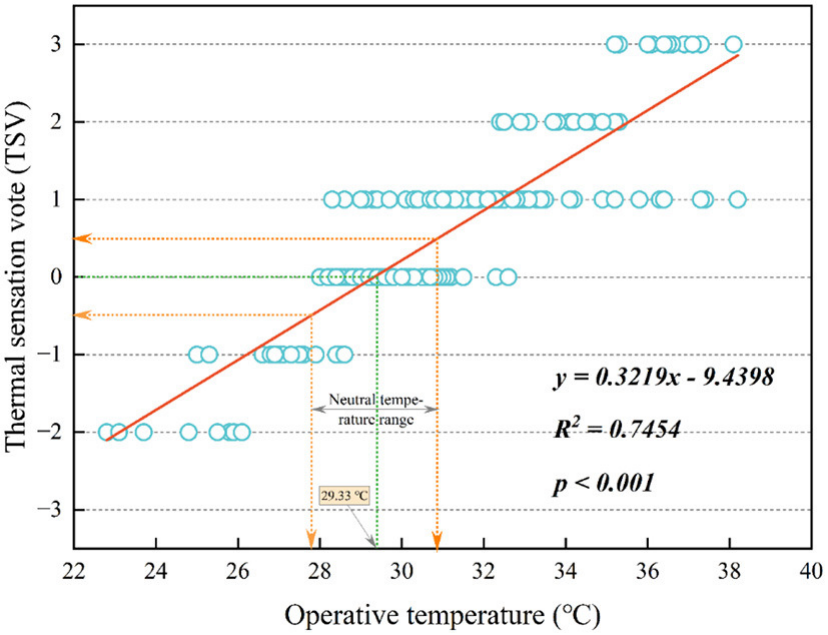
Results of the linear regression between TSV and *T*_*op*_.

*T*_*op*_ is an index for assessing outdoor thermal comfort. However, its applicability in the HSCW region still needs further study. Figure 10 shows that TSV was expressed as the sensitivity of respondents to indoor *T*_*op*_. The scatter plots of *T*_*op*_ and TSV were plotted, and their regression equation was shown as follows:


$$\begin{array}{l} TSV=0.3219\times T_{op}-9.4398\\ \;(R^{2}=0.7454,p<\;0.001) \end{array}$$

In summer, the TSV increases with advancing operative temperature. The neutral temperature of the respondents in summer can be calculated by setting the TSV to 0. We found that the neutral temperature was 29.33°C. By calculating the values of TSV equaling −0.5 and 0.5, the neutral temperature range of the respondent in summer can be calculated. We found that the neutral temperature range was 27.77–30.88°C.

### Thermal adaptive behavior

In our questionnaire survey, multiple-choice questions were posed to the respondents. Their adaptive behaviors in the summer included but were not limited to drinking cold beverages, changing clothes, using air-conditioners, using an electric fan, and using a hand-cranked fan.

**Figure 11:**
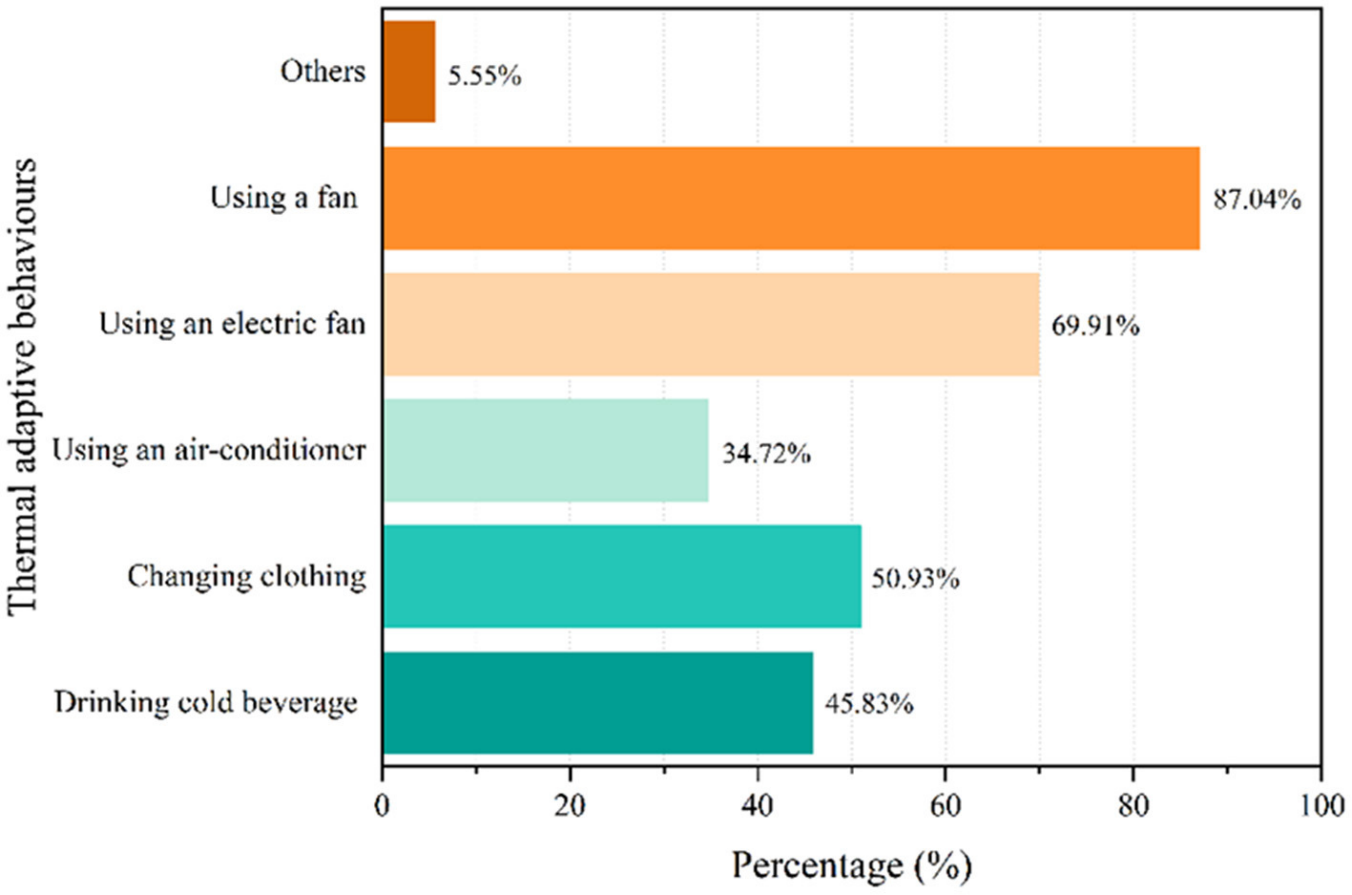
Results of thermal adaptive behavior voting.

Figure 11 shows the results obtained from the survey. Most respondents used hand-cranked fans to keep themselves comfortable. Other strategies include using electric fans, changing clothing, and drinking cold beverages, which are cheap and convenient. Much fewer residents used air-conditioners. They switch on it only when the indoor thermal environment is intolerable during the hottest hours rather than using it all day. Most residents agreed that air-conditioners consume a considerable sum of energy and are expensive to use. Thus, rural dwellers in Linshui tend to adopt conventional thermal adaptation behaviors rather than cumbersome and costly strategies.

In contrast with urban residents, who heavily rely on energy-consuming active cooling equipment, rural dwellers prefer traditional cooling strategies (e.g., *using a fan*). Besides the consumption value “frugality,” energy poverty (51) and low income (52) make rural dwellers reluctant to pay much for cooling. We hope that mounting studies on rural dwellers' strategy of using air-conditioners for cooling will appear in recent years and that the differences in health effects between using traditional cooling strategies and air-conditioners can be deeply investigated.

## Discussion

### Comparison of the neutral temperature

We decided to compare the neutral temperature obtained from this study and those presented in studies on indoor thermal comfort based on other regions and building types (see Table 5). The neutral temperatures obtained from the TSV and *T*_*op*_ linear regression equations differed by region and building type. The identified neutral temperatures in summer were markedly different from the results of other studies in the same climate region (i.e., HSCW region). Such divergent results may be due to differences in building types. Moreover, studies based on the HSCW region were conducted in urban areas, and their sites for analysis may be in an environment where the air-conditioner operates. However, this study was conducted in rural areas, and the entire dwelling was under natural ventilation during the study period.

**Table 5:** Comparison of this study and other studies.

**Region**	**Climate region**	**Type**	**Regression equation**	**Neutral temperature**	**Reference**
Xi'an	C	University building	*TSV* = 0.289 × *T*_*op*_−7.569	26.19°C	(53)
Guangzhou	HSWW	University building	*TSV* = 0.3596 × *T*_*op*_−9.5088	26.44°C	(54)
Guilin	HSCW	Urban dwelling	*TSV* = 0.2576 × *T*_*op*_−6.2295	24.18°C	(55)
Changsha	HSCW	University building	*TSV* = 0.33 × *T*_*op*_−8.61	26.09°C	(56)
Shihezi	SC	University building	*TSV* = 0.59 × *T*_*op*_−16.58	28.10°C	(57)
Changsha	HSCW	Office building	*TSV* = 0.3873 × *T*_*op*_−10.0962	26.07°C	(58)
Linshui	HSCW	Rural dwelling	*TSV* = 0.3219 × *T*_*op*_−9.4398	29.33°C	This study

Neutral temperatures can be similar in different climate regions, such as 26.19°C in Xi'an (a cold region) (53), 26.44°C in Guangzhou (an HSWW region) (54), and 26.09°C (56) or 26.07°C (58) in Changsha (an HSCW region). This outcome suggests that the thermal comfort of residents in the indoor environment was affected by regional differences marginally but by the indoor temperature with the air-conditioner operation considerably. This finding helps determine the appropriate air-conditioner temperature and reach two objectives jointly: (1) the vast majority of people reach a relaxed state, and (2) air-conditioner energy consumption can be minimized (59, 60). This finding also calls for further studies in rural areas. Dwellings in the rural areas of China need to be studied in-depth to discover a broader range of universal laws to enhance the comfort and quality of life of rural dwellers, especially in the post-pandemic era (61, 62).

### Limitations

This study used a field research method combining objective measurements of indoor thermal environment parameters and subjective questionnaire surveys. A typical indoor thermal survey was conducted in a rural dwelling in the HSCW region of China, where local thermally neutral temperatures were obtained. Although some interesting results were obtained, this study is by no means beyond reproach. Some research limitations are as follows. First, indoor thermal comfort is strongly influenced by season. This study was conducted only in the summer. Other seasons can be studied to comprehensively understand the changes in indoor thermal comfort throughout the year. Second, this study did not consider the effect of subjective factor differences (thermal resistance of clothing and activity status) on the thermal comfort of rural dwellers. These factors play an important role in shaping the thermal comfort of rural dwellers. Third, the field observation period is short in this study. We agree that it can be substantially expanded. Fourth, the number of respondents was too small to support the thermal comfort analysis of different ages. Last, the generalizability of the results may be questioned and challenged because we selected only one rural dwelling as the case study due to limited experimental conditions, such as the measurement equipment and the number of researchers.

## Conclusions

This study conducts indoor thermal environment measurements and subjective questionnaires in a rural dwelling in southwest China. It assesses human thermal comfort using operational temperatures, explores the thermal comfort conditions of rural dwellers in summer, and determines the local neutral temperature for indoor thermal comfort. The main findings of this study are summarized as follows.

1. In dwellings in rural areas, the presence of the window and its size are essential factors that affect the comfort of rural dwellers indoors. In summer, solar radiation through glazing can heat indoor space. Additionally, solar radiation directly affects the temperature and humidity, thereby influencing indoor thermal comfort.

2. Among the various meteorological parameters of the indoor thermal environment, residents in rural areas sensitively perceive the change in temperature and air velocity but have difficulty perceiving the change in humidity (the percentage of “no change” is 93.05%). This finding suggests that when people are exposed to a stable humidity environment (from 70 to 85%) for a long time, their ability to perceive that parameter seems compromised. That is to say, their adaptation to high humidity is evident in this region with the hot and humid summer.

3. Rural dwellers have developed considerable experience adapting to the local climate despite their poor economic conditions. The neutral temperature (29.33°C) in *T*_*op*_ is higher than the ASHRAE standard and the standard for urban residents in summer. Due to their long-term thermal adaptation effect, rural dwellers are also less thermally sensitive than urban residents and have an extensive acceptable range of indoor neutral temperatures (27.77–30.88°C).

4. In addition to psychological thermal adaptation, the adaptive behavior adopted by the rural dwellers significantly enhances their ability to tolerate adverse environmental conditions (the percentage of “comfortable” and “slightly comfortable” combined is 89.35%). This study suggests that these rural dwellers have developed unique lifestyles and measures to adapt to the harsh thermal conditions of summer. In addition, even when air-conditioners are installed in conventional homes, the influence of residents' previous cooling experience remains, which significantly increases their satisfaction with their environment. Thermal adaptation behavior alleviates residents' discomfort caused by temperature deviations and expands their comfort zone.

5. At this stage, many rural dwellers are older adults who still adhere to using very few or no energy-consuming cooling devices. They refuse to use the air-conditioner for space cooling even in the presence of high temperatures. This is dangerous because the high temperature may affect the health of older adults and thus lead to heat-related morbidity and even mortality. Therefore, the renovation program of residential buildings in rural areas should be further improved, and more passive cooling measures should be taken to enhance the indoor thermal environment, which can decrease energy consumption and energy bills on the one hand and ensure the health of residents in the extreme hot events on the other hand.

## Data availability statement

The raw data supporting the conclusions of this article will be made available by the authors, without undue reservation.

## Author contributions

DW: conceptualization, formal analysis, methodology, and writing—original draft. GZ: validation, and writing—review and editing. SL: validation and writing—review and editing. LY: conceptualization, funding acquisition, supervision, and writing—original draft. All authors contributed to the article and approved the submitted version.

## Funding

This research was supported by the Research Fund from Sichuan Rural Community Governance Research Center (No. SQZL2022B01).

## Conflict of interest

The authors declare that the research was conducted in the absence of any commercial or financial relationships that could be construed as a potential conflict of interest.

## Publisher's note

All claims expressed in this article are solely those of the authors and do not necessarily represent those of their affiliated organizations, or those of the publisher, the editors and the reviewers. Any product that may be evaluated in this article, or claim that may be made by its manufacturer, is not guaranteed or endorsed by the publisher.
